# Rapid expression and purification of the hepatitis delta virus antigen using the methylotropic yeast *Pichia pastoris*

**DOI:** 10.1186/s13104-017-2692-8

**Published:** 2017-07-27

**Authors:** Stephanie P. Cartwright, Roslyn M. Bill, Bui Tien Sy, Hieu Tran-Van, Hung Minh Nguyen

**Affiliations:** 1 School of Life & Health Sciences, Aston University, Aston Triangle, Birmingham, B4 7ET UK; 2 Faculty of Biology and Biotechnology, University of Science, Vietnam National University, Ho Chi Minh City, Viet Nam; 3 Department of Molecular Biology, 108 Military Central Hospital, 1 Tran Thanh Tong, Ho Chi Minh City, Viet Nam; 4 Center for Molecular Biology, Institute of Research and Development, Duy Tan University, K7/25 Quang Trung, Da Nang City, Viet Nam

**Keywords:** Hepatitis delta virus, HDAg, *Pichia pastoris*, Protein expression

## Abstract

**Objective:**

Patients with dual hepatitis B (HBV) and hepatitis D (HDV) virus infection are at an increased risk of progression to liver cirrhosis and hepatocellular carcinoma than patients with a single viral infection. Treatment of viral hepatitis due to dual HBV/HDV infection represents a challenge. Currently there is no vaccine against HDV. Recombinant production of HDV antigen (HDAg) is the first step towards a potential vaccine candidate and the development of assays for HDV detection.

**Results:**

This study demonstrates the expression of one HDAg isoform, S-HDAg, in *Pichia pastoris*. A recombinant vector carrying a tagged gene encoding S-HDAg under the control of the methanol-inducible promoter *AOX1* was designed and integrated into *P. pastoris* X33. The protein, which was purified using a Ni^2+^ affinity column and eluted at 100–150 mM imidazole, has potential as a recombinant antigen for further study.

## Introduction

It is estimated that ~240 million people are chronic hepatitis B surface antigen (HBsAg) carriers, of which ~15–20 million are also infected with hepatitis delta virus (HDV) [1–3]. The HDV virion comprises an RNA genome, a single HDV-encoded antigen (HDAg) and a lipoprotein envelope provided by HBsAg [4–7]. HDAg comprises two isoforms, small HDAg (S-HDAg) and large HDAg (L-HDAg) [8, 9]. These two isoforms share the same core sequence, but L-HDAg is extended by an additional 19 amino acids at the carboxyl terminus of S-HDAg. S-HDAg may represent a candidate for human vaccine development. Protection induced by immunization of adjuvanted S-HDAg (p24) was evaluated in woodchucks challenged with HDV by measuring humoral- and T cell-mediated responses to HDAg [10]. In another study, a DNA vaccine expressing S-HDAg generated a higher titer of anti-HDV antibodies than one expressing L-HDAg [11]. However, efforts to characterize and evaluate the immunological properties of S-HDAg have been limited due to the lack of proper methods for efficient expression and purification of S-HDAg. In this work, we present a short procedure to express and detect S-HDAg in *Pichia pastoris* culture medium.

## Main text

### Methods

#### PCR amplification of the *S*-*HDAg* gene

Two primers, HDAg-F: 5′-GCTCTAGATTTGGGAATCCCTGGTTTCC-3′ and HDAg-R: 5′-GCGGTACCATGAGCCGGTCCGAATCG-3′ (*Xba*I and *Kpn*I sites underlined, respectively), were used to amplify the *S*-*HDAg* gene. The volume of the PCR reaction was 50 µL including: 1× Phusion buffer, 0.2 mM dNTP (NEB, N0446S), 0.5 mM each primer (IDT) and 5 ng pHDV3 plasmid as a template, 1U Phusion High-Fidelity DNA polymerase (NEB, M0530S). The PCR reaction was performed by using the following program: 98 °C for 30 s; 30 cycles of (98 °C for 10 s, 55 °C for 30 s, 72 °C for 30 s) and final extension at 72 °C for 5 min. The PCR product was analyzed by electrophoresis using a 1% (w/v) agarose gel (BioBasic, D0012) and visualized by Red-Safe Solution (iNtRON, 21141) on a Blue LED Illuminator. The desired DNA band ~590 bp was excised from the gel and purified by QIAquick Gel Extraction Kit (Qiagen, 28706) following manufacturer’s instructions.

#### Cloning of the *S*-*HDAg* gene into pPICZαA

##### Enzymatic digestion and ligation

The purified *S*-*HDAg* gene and vector pPICZαA (TFS, V19520) were digested with *Xba*I and *Kpn*I (NEB, R0145S and R0142S, respectively) and purified by QIAquick PCR Purification Kit (Qiagen, 28106) following the manufacturer’s instructions. The digested *HDAg* gene was ligated into the linearized vector pPICZαA using T4 DNA ligase (NEB, M0202S). The reaction was performed in a 20 µL volume including 2 µL 10× Rapid Ligation Buffer, 8 µL DNA (~100 ng), 1 µL 5 U/µL T4 DNA ligase and incubated at 22 °C for 2 h.

##### Transformation and screening of *E. coli*

10 μL of the ligation mixture was transformed into competent *E. coli* DH5α cells by heat shock at 42 °C for 30 s. The cells were then recovered by adding 500 µL liquid LB medium and incubating at 37 °C for 1 h and then plated on LB plates supplemented with 25 μg/mL Zeocin (TFS, R25001). After incubated at 37 °C overnight, ten colonies were cultured in 3 mL liquid LB medium supplemented with 25 µg/mL Zeocin at 37 °C overnight. The recombinant plasmids were isolated from the cell pellets using a GeneJET Plasmid Miniprep kit (TFS, K0503) following the manufacturer’s instructions and digested using *Xba*I and *Kpn*I for screening positive plasmids carrying the *S*-*HDAg* gene.

#### Sequencing and analysis

In order to confirm positive clones, purified plasmid was used for nucleotide sequencing. 5 µL of eluted plasmid was subjected to cycle sequencing with 1.0 µL of the ABI Prism BigDye terminator cycle sequencing ready reaction kit (ABI) using 0.5 µL of 5′AOX-F: 5′-GACTGGTTCCAATTGACAAGC-3′ on the *AOX1* promoter and 3′AOX1-R: 5′-GCAAATGGCATTCTGACATCC-3′ on the *AOX1* terminator. Consensus sequences were generated by alignment of both sequenced strands after validation using DNAstar software V7.

#### Expression of recombinant S-HDAg in *P. pastoris*

##### Plasmid preparation

A positive colony of *E. coli* was cultured at 37 °C overnight in 50 mL liquid LB medium supplemented with 25 μg/mL Zeocin. The recombinant plasmid (pPICAαA-S-HDAg) was then isolated by GenElute Plasmid Midiprep kit (Sigma-Aldrich, NA0200) following the manufacturer’s instructions and linearized using *Pme*I (NEB, R0560S). The linearized plasmid was then separated on a 1.5% agarose gel and purified by Wizard SVGel and PCR Clean-Up System (Promega, A9281) following the manufacturer’s instructions.

#### Transformation and screening of yeast

##### Transformation

5 μg of the linearized recombinant vector was transformed into 50 µL of competent *P. pastoris* X33 or SMD1163 cells using a Gene-pulser electroporator (Bio-Rad) at 1800 V (25 µF, 600 Ω) in a 10 mm gap electroporator cuvette. After adding 1 mL 1 M ice-cold sorbitol, the cells were recovered at 30 °C for 2 h. 100 µL of the transformation mixture were then plated on YDPS plates supplemented with 100, 500 and 1000 µg/mL Zeocin and then incubated at 30 °C for 2–3 days until colonies appeared.

##### Induction on a small scale

For expression screening, 24 colonies of each parent strain, X33 or SMD1163, were cultured at 30 °C in 2.5 mL liquid BMGY media without Zeocin in a Micro-24 plate (Corning) to A_600_ around 15–20. Cells were centrifuged and transferred to 10 mL induction BMMY medium. Cells and culture media were harvested every 24-h post-induction. 1% methanol was added every 24-h post-induction.

##### Secreted protein preparation

Cultures were centrifuged at 5000 rpm for 3 min and 20 µL of the supernatant was taken forward for immunoblot analysis.

##### Intracellular protein preparation

Cell pellets were used to determine total, intracellular protein. 1 mL breaking buffer (50 mM Na_2_HPO_4_, 50 mM NaH_2_PO_4_, 2.0 mM EDTA, pH 7.4, 100 mM NaCl and 5% glycerol; pH 7.4), 2.0 µL protease inhibitor (Calbiochem) and 200 mg glass bead were added to the cell pellets. The cells were then lysed by breaking at 50 Hz for 3 min in a Tissue Lyser LT (Qiagen). The supernatant was transferred to a 1.5 mL Eppendorf and centrifuged at 13,000 rpm for 15 min. The cell lysate was used for immunoblot analysis.

##### Immunoblot

For detection of the HDAg-His-tag fusion protein, immunoblotting was used to detect the His_6_-tag fused to the HDAg protein in the supernatant (culture medium) or intracellular protein (cell lysate). 20 µL (10 µg) of each sample and 5 µL Protomarker pre-stained protein ladder (National Diagnostics) (10–225 kDa) were applied onto a 12.5% SDS gel and run in 1× Tris/glycine/SDS (GeneFlow) at 100 V for 1 h. The SDS gel was transferred on to a nitrocellulose membrane (Whatman, 09-301-111), blocked in 5% milk in 1× PBS buffer and incubated with primary antibody (6× His monoclonal antibody (Serotec) at a 1:5000 dilution at room temperature for 1 h). After washing with 1× PBST, the membrane was incubated with secondary antibody against mouse IgG conjugated with HRP (Sigma, A0545) at a 1:5000 dilution for 1 h. After washing with 1× PBST, protein bands on the membrane were detected using EZ-ECL chemiluminescence solution (Geneflow, 20-500-120) and visualized using a Uvitec instrument.

#### Nickel affinity purification

Recombinant protein was purified a using a His-trap column. Total secreted protein from 300 mL culture broth was dialyzed against binding buffer (300 mM NaCl, 10 mM imidazole, 50 mM NaH_2_PO_4_, pH 8.0; Sigma-Aldrich, 56750) which was also used as the binding and equilibration solution. A 5 mL His-trap HP column (GE Healthcare) was equilibrated with 5 column volumes of binding buffer. All dialyzed protein (5 mg) was loaded into the column with a flow rate of 1 mL/min for 50 min. The column was washed with 5 column volumes of binding buffer followed by 5 column volumes of wash buffer (300 mM NaCl, 30 mM imidazole, 50 mM NaH_2_PO_4_, pH 8.0). The protein was eluted with elution buffer (300 mM NaCl, 250 mM imidazole, 50 mM NaH_2_PO_4_, pH 8.0) at a flow rate of 1 mL/min for 20 min. Each 1 mL fraction was analyzed by SDS-PAGE and visualized using a silver staining kit (Sigma-Aldrich). The protein concentration of the eluted fractions was quantified using a Bradford kit (BioBasic).

### Results

#### Cloning and sequencing

**Fig. 1:**
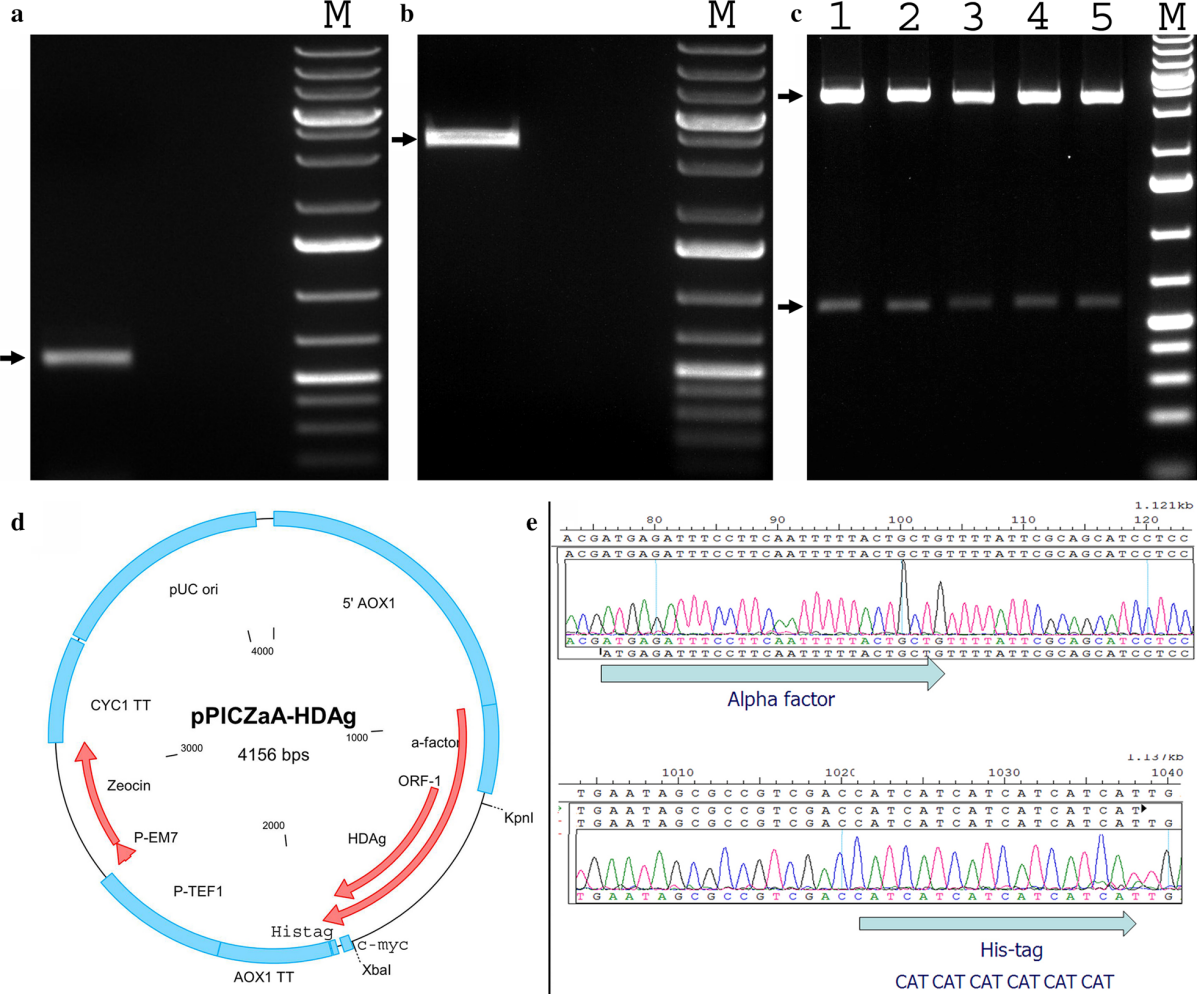
Cloning of the *S*-*HDAg* gene in the vector pPICZαA. **a** An agarose gel image showing the PCR product. The *arrow* indicates the position of the PCR product. **b** An agarose gel image showing the vector pPICZαA. The *arrow* indicates the position of the vector pPICZαA. **c** An agarose gel image showing the recombinant vector pPICZαA-S-HDAg isolated from five *E. coli* colonies (*lanes 1–5*) cut by *Xba*I and *Kpn*I. **d** Diagrammatic map of the recombinant vector pPICZαA-S-HDAg (drawn using the Clone Manager Suite). **e** A partial sequence of the recombinant vector pPICZαA-S-HDAg including the α-factor, the *S*-*HDAg* gene, and His_6_-tag (using DNA star software). The *arrows* indicate the positions of the vector pPICZαA and the S-HDAg gene, respectively. *M* GeneRuler 1 kb plus DNA ladder (Fermentas)

A 589 bp fragment comprising the *HDAg* gene was amplified by PCR (Fig. 1a). The PCR amplicon was cloned into the vector pPICZαA by enzymatic digestion and ligation. To confirm this, the recombinant vector pPICZαA-S-HDAg was digested with *Xba*I and *Kpn*I. Two bands of 589 and 3567 bp in length were produced as expected (Fig. 1b, c). To ensure the *S*-*HDAg* gene was in frame, two primers 5′AOX1-F and 3′AOX1-R were used for sequencing a segment of the recombinant vector pPICZαA-S-HDAg (Fig. 1d). As shown in Fig. 1e, the *HDAg* gene was cloned into vector pPICZαA and located between the α-factor at the 5′-end and the hexa histidine-tag at the 3′-end. The linearized recombinant vector pPICZαA/S-HDAg was transformed into freshly-prepared competent *P. pastoris* X33 or SMD1163 cells. Positive colonies were screened based on their resistance to Zeocin due to expression of the Zeocin resistance gene.

#### Protein expression and purification

**Fig. 2:**
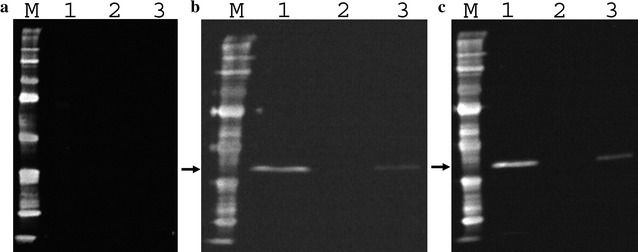
Expression of the S-HDAg-His_6_-tag fusion protein. An SDS-PAGE gel image showing the expression of the S-HDAg-His_6_-tag fusion protein after **a** 24-h, **b** 48-h and **c** 72-h induction. *Lanes 1–3* indicate three colonies, of which *lanes 1* and *3* show recombinant protein expressed after 48-h and 72-h induction; *M* are Protomarker Protein Markers (National Diagnostics). Protein was probed with a 6× His monoclonal antibody (Serotec)

**Fig. 3:**
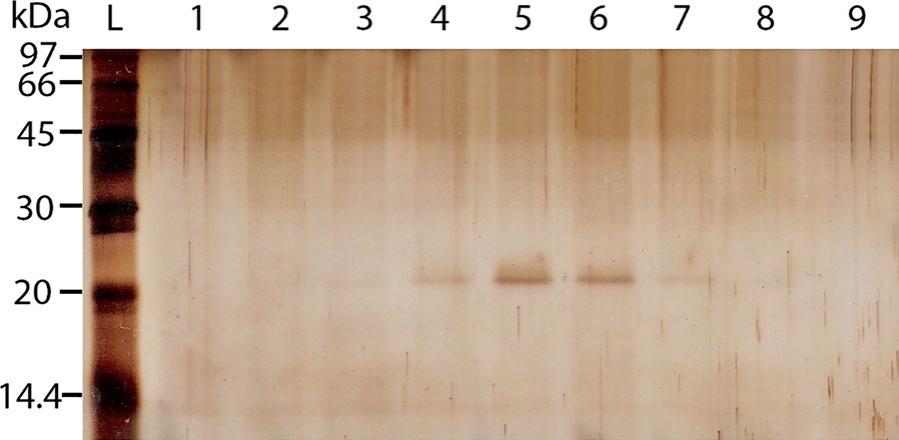
Purification of S-HDAg-His_6_-tag fusion protein using nickel affinity chromatography. *L* indicates protein ladder; *1–9* are eluted fractions which have imidazole concentrations ranging from 72 to 156 mM (the concentration interval between 2 consecutive fractions is about 12 mM). The gel was visualized by silver staining

To test the expression levels of the HDAg-His-tag fusion protein, 24 positive colonies of each yeast strain were cultured in 2.5 mL BMGY media in a Micro-24 microplate and transferred into 10 mL BMMY supplemented with 1% v/v methanol as an inducer at three time points: 24, 48 and 72 h of induction. Under the control of the promoter *AOX1* (methanol inducible promoter), the *S*-*HDAg* gene was expressed in *P. pastoris* X33 but not in SMD1163. For the X33 strain, the expressed protein signal was detected in the culture medium after 48 and 72 h of induction, while no signal was detected in the cell lysate at all three induction time points. A clear band at 25 kDa was observed by immunoblot (Fig. 2). This is the expected size of the recombinant protein including α-factor, S-HDAg, c-myc epitope, and His_6_-tag. The recombinant protein was purified exploiting its fused His_6_-tag. The protein eluted at imidazole concentrations from 108–144 mM (wells 4–7, Fig. 3), but not at other concentrations (data not shown). This protein had a molecular weight of 25 kDa which is similar to the predicted molecular weight of the recombinant protein. The yield of purified protein was 115 µg/L culture medium.

### Discussion

S-HDAg may present a good candidate for HDV vaccine development and for diagnostic assays of HDV, but its characterization and immunological evaluation are still limited. One reason is that expression and purification are not effective [12]. For example, expression of the S-HDAg protein has been performed in several cells including *E. coli* [13, 14] and baculovirus/insect cells. However, the former lacks the systems for post-translational modifications and the latter results in rapid degradation of the HDAg protein after 2 days post-infection [12, 15]. The insertion of the *HDAg* gene into the chromosome of animal cells resulting in a stable cell-line is a good choice, but this is yet to be reported because HDAg is a nuclear protein and the accumulation of this protein results in significant cytotoxicity. In 1990, a number of HDAg-positive HeLa clones were developed, but these cells were lost in culture, whereas a proportion of HDAg-positive HepG2 clones were expanded successfully [16], suggesting that HDAg cytotoxicity may contribute to the cytopathic nature of HDV that was postulated previously [17]. Transient expression in mammalian cells mediated by viral systems (e.g. vaccinia virus) may be possible as well, however these viruses result in cell death and lysis.

Post-translational modifications have been demonstrated to participate in modulating properties and functions of several proteins [18, 19]. HDAg has been identified as being post-translationally modified, which is important for its RNA replication and cellular localization [20]. Yeast expression systems in general, and in particular *P. pastoris*, have several advantages such as being able to perform eukaryotic post-translational modifications. In this study, the HDAg antigen was expressed for the first time in *P. pastoris* and secreted into the culture medium, which aids in purification of the protein. This preliminary finding will aid further studies of the S-HDAg protein.

## Limitations

Our study had some limitations. We examined the expression of only one HDAg isoform, S-HDAg, in *P. pastoris*. We also did not address scale-up of the recombinant protein. Finally, we did not characterize the immunological properties of the purified recombinant HDAg protein.

## Abbreviations

BMMY: buffered methanol-complex medium
BMGY: buffered glycerol-complex medium
*E. coli*: *Escherichia coli*
HDAg: hepatitis delta antigen
LB: Luria Broth
MCS: multiple cloning sites
YPD: yeast extract peptone dextrose
